# Eight Cases of Syphilis of the Finger in Medical Men

**Published:** 1882-03

**Authors:** Fessenden N. Otis

**Affiliations:** Clinical Professor of Diseases of the Genito-Urinary System, College Physicians and Surgeons, Attending Physician Charity Hospital, Consulting Surgeon St. Elizabeth Hospital, etc.; New York


### CLINICAL REPORTS.

### EIGHT CASES OF SYPHILIS OF THE FINGER IN MEDICAL MEN.

Reported by FESSENDEN N. OTIS, M. D.

Clinical Professor of Diseases of the Genito-Urinary System, College Physicians and Surgeons, Attending Physician Charity Hospital, Consulting Surgeon St. Elizabeth Hospital, etc.

Case I.—W. N., M. D., aet. 26, was in good general health up to three weeks ago. In the latter part of September, 1881, he noticed a small, red papule on the superior surface of the forefinger, at the middle of the second phalanx. He had been for over a month on the venereal service of Charity Hospital. He had never noticed any previous abrasion at the point of appearance of the papule. He is not aware of having had any special exposure of this finger. He had been in the habit twice a week of making vaginal examinations of venereal patients. The papule was painless, had a pale red color and a slight boggy feel but was without distinct induration. Thinking it might contain a splinter, an incision was made into it but no splinter was found; no pus, only blood, escaped. This cut remained open and assumed the form of a small ulcer with sharply cut edges, 3-16 of an inch in diameter, and 1-16 of an inch in depth, circular, with smooth, shiny, red floor. This exuded a secretion which accumulated, dried and formed a scab which dropped off at the end of twenty-four hours, with an escape of 3 or 4 drops of seropurulent fluid. It would then exude, dry, and scab over again. I examined it about the 10th of November, when exudation first commenced, and detected in connection with it, an enlarged and somewhat tender gland in the axilla. Several days after I found an enlarged epitrochlear gland in the right arm. A deep red areole with a scaly border, now surrounded the lesion. Patient's health was good up to three weeks ago (or six weeks after the discovery of the papule) when without apparent cause he began to suffer with headache and general malaise. Insomnia well marked; appetite pretty fair. He, however, kept about his work at the hospital; he had some febrile excitement; temperature about 100 in the evening. These symptoms all disappeared in about ten days, and he returned to his general health, and was feeling perfectly well, when on December 18, looking, as had for some time been his habit on retiring, he discovered on his body a distinct eruption which he described as papular in character.

Examination at the present time (about eleven weeks' from the discovery of the original lesion) shows a discrete eruption of papules both fine and coarse, scattered over the body, most prominent on the chest and arms, and pale red in color; also distinctly indurated glands in cervical, epitrochlear and inguinal regions, characteristically enlarged, and one also in right axilla enlarged and tender. The throat is congested, a single scab is found in the hair. The patient, who had been desirous of waiting until the diagnosis of syphilis was absolutely certain, was now put upon a systematic treatment for that disease.

Case 2.—1878, S. S. B.; presented with a papule of the middle finger of right hand, about the size of a silver three cent piece, just over the second joint, elevated and non-suppurating. It appeared as a red spot about two weeks' previous, and has gradually become elevated and with no distinct induration. About six days ago a dry scale appeared in the centre and a molecular necrosis started from that joint. He has poulticed it for the last week. There is no local tenderness but some pain in the arm stretching up from the lesion as far as the elbow. A single enlarged gland is found in the corresponding axilla about the size of a filbert.

The patient was advised that the lesion was probably syphilitic and instructed to wait for signs at other points. In this case there was no positive induration about the lesion, only a boggy feel. The patient has no idea of any date of exposure. He attended a confinement case on April 6, but had no suspicion of syphilis in the case.

I lost sight of this patient until July, 1881, when I was informed by Dr. E. F. Ward, of New York, that he subsequently had roseola and a papular eruption developed, and that he was at this time suffering from hemiphlegia which had come on suddenly.

Case 3.—In latter part of December, 1871, the patient, a physician noticed a red spot upon the dorsal surface of the right index finger, near the base of the second phalanx. The spot when noticed was about an inch in diameter and continued slowly to increase in circumference and to become raised until within three weeks it reached nearly the size of a three cent piece and looked precisely like a vaccine vesicle without a central depression. It soon became incrusted, but by the application of poultices the crust was removed, leaving a well rounded ulcer about one-third of an inch in diameter, excavated, clean, without discharge, the edges raised and all of a deep red color and sluggish in appearance, neither inclining to heal itself nor to yield to treatment. The base was boggy and no induration whatever could be discovered, although searched for by a distinguished surgeon in this city, and by him the lesion was confidently pronounced to be at most a *simple* chancre. Another surgeon familiar with syphilis was equally confident of its simple character. A third who saw it while a small papule, regarded it with suspicion, and advised the patient to consult some surgeon who gave especial attention to such cases. The patient then came to me. My opinion was strongly in favor of a syphilitic origin for the lesion, but the patient desired to wait for further proof before commencing constitutional treatment. The ulcer showed no sign of improvement. The extended finger was bandaged to a splint, rendering the point immovable, and allowed to remain so two weeks, but without improvement. I then advised the application of iodoform powder. Within forty-eight hours a decidedly favorable change had taken place, and within ten days the ulcer was perfectly healed. Once or twice afterward the skin was accidentally broken, but on reapplying the iodoform, it healed kindly. From the first appearance of the spot till the healing of the ulcer, no pain or discomfort was felt. After some four months, that is to say, in the following April, the doctor called to inquire about an eruption which had made its appearance a week or two previously upon his breast and arm chiefly, sparsely on his face and head, which was quite bald. The eruption was of a dull red color, slightly elevated, and several papules were encircled by a line of exfolitating epidermis. They were free from itching and were discovered by the accident of their apperanace on the face and scalp, as they caused no sensation and were not preceded by any fever, headache or other constitutional disturbance. Examination showed distinct gland enlargements in the cervical, inguinal and epitrochlear regions. He was then for the first time, put on a regular mercurial course, viz: one pill of mass. Hydrarg, 2 gr. combined with 1 gr. of the exsiccated sulphate of iron, 3 times a day. At about this time this patient's wife began to complain of a profuse vaginal discharge, having been previously in good health, and free from any leucorrhœal trouble. About three months subsequent to this, a characteristic papular eruption appeared on her face and body, general gland enlargements distinct and prominent in groin, neck and epitrochlear regions. She too was then put on a systematic mercurial course similar to that of her husband. Both were kept under treatment for about a year and a half, when no signs of syphilitic trouble having appeared for several months, it was discontinued. To-day, Feb. 21, 1882, the doctor reporting by my request, states that now nearly ten years from the disappearance of the disease, and the cessation of all treatment, both he and his wife have been and are now free from any evidence of syphilis.

Two other cases of the occurrence of syphilis in physicians where the initial lesion was situated on the right forefinger have been reported to me during the present winter, and in addition to these I am cognizant of three other cases in New York City, two gynecologists and one distinguished surgeon, who have had syphilis through an initial lesion of the finger.

The first point of interest in considering the foregoing cases, is the danger to which any physician who treats diseases of females or attends females during the parturient condition, is more or less exposed, and the necessity of using extraordinary precautions in examining or attending every case to which a suspicion of syphilis could possibly attach and habitually to protect by previous application of elastic collodion any cracks or abrasions about the nails or joints of the fingers, especially of the right forefinger, and to use a lotion of carbolic acid (1 to 100) or of the liquor potassa permanganatis, 1 part to 40 of water, as a habit after all digital examinations of the female genital apparatus.

It may be safely asserted that a pre-existent abrasion or fracture of the skin or mucous membrane, is absolutely essential to the acquirement of syphilis; and that in any case when syphilis has been acquired without the recognition of a local initial lesion, it has been present, but overlooked. Destruction of tissue is not essential to the perfect initial lesion of syphilis.

Healing of an abrasion may take place after an inoculation, just as promptly and as perfectly as if no inoculation had taken place, and the point of induration following may be so small and insensitive, that it would easily escape observation.

In case of the wife of physician (case III), the initial lesion was not discovered. Her eruption was only preceded by a profuse vaginal discharge. There was never any recognized open lesion on the penis of her husband. It might be said of her that the inoculation had taken place through the influence of the semen. Mireir, of Marseilles has made repeated experiments of inoculating the semen of a person in the active stage of syphilis, upon healthy persons, but without effect. It is more probable, in fact, almost a certainty, that the disease in this case was acquired from a syphilitic papule, of which there were at one time several on the penis. Abrasion occurring during coition coming in contact with an abrasion of the os or vaginal mucous membrane, might there establish the initial lesion, resulting in the vaginal discharge, which preceded the syphilitic eruption, which was the first recognized evidence of syphilis in the doctor's wife.

The second point of interest is in the uniform and characteristic physical appearances, presented in the initial lesion of syphilis of the finger, coming on always as a papule, coming soon to be of a deep red color, and presenting a superficial abrasion, becoming circular and deeper by a slow molecular necrosis; not by ulceration with formation of pus. The secretion thin and serous, and drying into a scab which is soon displaced by the fluid accumulating underneath.

The entire absence of induration: in its place a slight, flat, juicy-looking, boggy swelling, or elevation about like a small peppermint in size and thickness—early appearance of an enlarged and somewhat tender gland in the axilla of the corresponding side.

I would like to call attention to an interesting fact in regard to the efficacy of remedial measures, viz: that in five of the above mentioned cases, a careful systematic mercurial treatment was pursued during a period, varying from one and a half to two and a half years. Eight healthy children have been born, and both they and the parents have continued free from any evidence of syphilis up to this date.

*New York*, March 1, 1882.

